# ACCESS TO HOSPITAL-BASED PALLIATIVE CARE SERVICES FOR OLDER ADULTS IN IOWA

**DOI:** 10.1093/geroni/igac059.2519

**Published:** 2022-12-20

**Authors:** Hyesu Yeo, Elisa Childs

**Affiliations:** University of Georgia, Athens, Georgia, United States; The University of Georgia, Athens, Georgia, United States

## Abstract

**Background:**

U.S. Medicare covers many palliative services; however, Iowa’s rurality and high rate of older adults (OAs) aged 65 or over make it unclear whether older Iowans have equitable service access. Hospital-based palliative care services (HBPCSs) include curative treatment, whereas other providers of palliative care may not. Thus, this study only examined OAs’ geographic access to HBPCSs in Iowa.

**Methods:**

This study used the American Hospital Association and U.S. Census Bureau estimate in 2017. The geographical distribution of hospitals and HBPCSs was examined using a county-level approach, considering rural/urban status (using the Office of Management and Budget’s definitions) and OA population proportion.

**Results:**

Of the 99 counties in Iowa, 89 were rural. Of the 116 hospitals statewide, one urban county and nine rural counties had no hospital. A total of 52 hospitals provided HBPCSs in 43 counties across nine urban and 34 rural counties. HBPCSs were primarily located in central Iowa, with northern and southern Iowa having the lowest access to HBPCSs. The OA population ratio was higher in counties without HBPCSs (19%) than HBPCSs (15.7%). All 54 counties with over a 20% OA population were concentrated in rural counties, while only 37% of these rural counties had HBPCSs.

**Conclusion:**

This study suggests a regional imbalance of HBPCS providers for OAs in Iowa. Results showed that rural communities with a high ratio of OAs lack HBPCSs and suggest HBPCSs should be expanded at hospitals in northern and southern Iowa counties where many OAs live.

